# Adenosine 5’-triphosphate’s role in bradycardia and syncope associated with pulmonary embolism

**DOI:** 10.1186/s12931-018-0848-2

**Published:** 2018-07-28

**Authors:** Amir Pelleg, Edward S. Schulman, Peter J. Barnes

**Affiliations:** 1 Department of Medicine, Drexel University, College of Medicine, NCB, MS# 470, 245 N 15th Street, Philadelphia, PA 19102 USA; 2 Airway Disease Section, National Heart and Lung Institute, Imperial College, London, UK

**Keywords:** Vagus, P2X receptor, Cardio-cardiac reflex, Pulmonary-pulmonary reflex

## Abstract

Adenosine 5′-triphiosphate (ATP) is released from cells under physiologic and pathophysiologic conditions. Extracellular ATP acts as an autocrine and paracrine agent affecting various cell types by activating cell surface P2 receptors (P2R), which include trans-cell membrane cationic channels, P2XR, and G protein coupled receptors, P2YR. We have previously shown that ATP stimulates vagal afferent nerve terminals in the lungs by activating P2X2/3R. This action could lead to bronchoconstriction, cough and the local release of pro-inflammatory neuropeptides. In addition, ATP markedly enhances the IgE-dependent histamine release from human lung mast cells. Thus, we have proposed for the first time that extracellular ATP plays a mechanistic role in pulmonary pathophysiology in general and chronic obstructive pulmonary disease (COPD), and acute bronchoconstriction in asthma in particular. The present review examines whether ATP could also play a role in bradycardia and syncope in a subset of patients with pulmonary embolism.

## Background

For more than four decades it has been known that more than 10% of patients with pulmonary embolism (PE) manifest syncope and/or bradycardia [1]. The frequency of PE patients with syncope ranges from 1 to 40% with an average of 15% [2–6]. A recent multi-center prospective study, which employed guidelines-based clinical assessment of PE [7], has found that PE is identified in nearly one of every six patients hospitalized for a first episode of syncope [8].

According to the European Society of Cardiology and American Heart Association, PE is classified into three prognostic categories: Massive PE, sub-massive PE and non-massive PE. Patients with massive PE may present with severe symptoms including syncope, whereas those with non-massive PE may be asymptomatic or have limited symptoms. Three mechanisms have been proposed to explain PE-induced syncope [9, 10]: (i) Massive PE causing drop in cardiac output, hypotension, and reduced cerebral perfusion [1], (ii) A complete heart block in the setting of preexisting left bundle branch block [11], and (iii) A central vagal reflex [12] triggered by the stimulation of vagal mechano-sensory nerve endings in the left ventricle caused by right atrial stretch-dependent increased sympathetic input to the heart in the setting of decreased left ventricular filling [10]. A more recent study in PE patients have found that right ventricular (RV) dysfunction and saddle embolism were more prevalent in those patients with syncope compared to those without [13]. However, it was not determined whether either RV dysfunction or saddle embolism were just associated with syncope or one of its causes.

Adenosine 5′-triphosphate (ATP) is found in every cell of the human body where it plays a major role in cellular metabolism and energetics. ATP is released from cells under physiologic and pathophysiologic conditions [14]; extracellular ATP acts as an autocrine and paracrine agent before it is rapidly degraded to adenosine by ecto-enzymes (mainly CD39 and CD73). The effects of extracellular ATP are mediated by P2 cell-surface receptors (P2R), which include trans-cell membrane cationic channels, P2XR, and G-protein coupled receptors, P2YR. Various cells types in the airways express P2R [15].

We have previously shown that ATP stimulates vagal afferent nerve terminals in the lungs by activating P2X2/3R [16, 17]. This action could lead to bronchoconstriction [18, 19], cough and the local release of pro-inflammatory neuropeptides (see below). In addition, ATP markedly enhances the IgE-dependent histamine release from human lung mast cells [20]. Furthermore, we have shown that P2YR mediates ATP-induced increase of [Ca^+ 2^]_i_ in eosinophils [21], which is associated with the chemotactic action of platelet-derive ATP on eosinophils [22]. Based on these early studies in vitro and in vivo, we have proposed in 2002 for the first time that extracellular ATP plays an important mechanistic role in pulmonary disorders including PE [23]. Since then, numerous studies have yielded voluminous data supporting this hypothesis [24].

Here we consider the putative mechanistic role of extracellular ATP in bradycardia and syncope associated with PE.

## ATP-triggered vagal reflexes

Since the early 1940s is was known that extracellular ATP could increase vagal input to the heart, which is manifested in negative chronotropic and dromotopic effects (for a reviews see [25, 26]). The mechanism of vagal recruitment by ATP constitutes a cardio-cardiac central vagal reflex that is triggered by the activation of P2X2/3R localized on vagal sensory nerve terminals in the inferior-posterior wall of the left ventricle [27]. Similarly, extracellular ATP stimulates vagal sensory nerve terminals in the lungs by the activation of P2X2/3R localized on C and Aδ fibers [16, 17], thereby triggering a pulmonary-pulmonary central vagal reflex resulting in bronchoconstriction and increased intra-airways pressure [19]. In both heart and lungs, these vagal sensory terminals are bimodal; i.e., they are sensitive to mechanical and chemical perturbations, e.g., stretch and ATP, respectively. In addition, it is highly likely that the stimulation of the vagal sensory nerve endings results also in an axonal reflex, leading to the local release of neuropeptides, including tachykinins and calcitonin gene-related peptide that have pro-inflammatory effects in the airways. Furthermore, there is a large body of evidence for ATP being a co-transmitter in most, if not all, peripheral and central neurons, therefore, the activation of an axon reflex by ATP probably results also in the localized release of ATP in a positive feedback-loop manner.

ATP-vagal interactions in the heart and lungs could have important clinical implications. In the heart, the cardio-cardiac vagal reflex triggered by ATP could be involved in vasovagal syncope as well as bradycardia associated with inferior-posterior left ventricular (LV) myocardial infarction. Indeed, it has been recognized that “reflex vagal activity, probably enhanced by local phenomena, plays an important role in bradyarrhythmias occurring after acute myocardial infarction” [28], and that atropine could be an effective treatment in this setting [28, 29]. The pulmonary-pulmonary central vagal reflex triggered by ATP could be involved in bronchoconstriction and cough associated with pulmonary inflammation [23, 24].

## Platelet activation, ATP and vagal stimulation

That abnormalities of ventilation are associated with platelet activation has been known for many years; it is manifested in various pulmonary pathophysiologic settings, which include massive platelet activation, such as in PE. Almost a century ago, Binger et al. reported that freezing of the vagus nerves converted the rapid shallow breathing associated with acute PE to slow deep breathing [30]. Since then, numerous studies in different animal models have implicated the vagus nerve not only in rapid shallow breathing but also in the bronchoconstriction observed during the early phases of PE (see e.g. [31]). Furthermore, in a rabbit model, lung irritant receptors contribute to the reflex hyperpnea and bronchoconstriction during PE [32]. In addition, platelet activating factor (PAF) has been implicated in the pathophysiology of asthma in general and bronchial hyperresponsiveness in particular (see e.g. [33]). Indeed, depletion of platelets prevents some of their pro-asthmatic effects (see e.g. [34]).

Platelets contain large amounts of ATP, i.e., in the molar range [35], a significant portion of which is released during platelet activation [36–38]. In addition to ATP, platelet activation is known to result in the release of a host of mediators, including serotonin and histamine [39, 40]. Both ATP and serotonin induce vagal-dependent bronchoconstriction in animal models [41], [19]. Thus, the massive activation of platelets in PE could result in the localized release of large amounts of ATP, which stimulates vagal sensory nerve ending and thereby, increased vagal input to the lung and heart.

## Role of ATP in syncope and braydycardia associated with PE

Based on the experimental data in animal models and observations in human subjects summarized above, it is tempting to speculate that extracellular ATP could play a mechanistic role in syncope and bradycardia associated with PE. The proposed mechanism consists of the following steps: (i) activation of platelets in the lungs, localized release of ATP, and (ii) ATP-triggered pulmonary-pulmonary and cardio-cardiac vagal reflexes.

Platelet activation, which is evident after acute PE, correlates with the severity of RV dysfunction [42]. That vasoactive mediators are released from activated platelets associated with PE has been known for a long time [43]; because large amounts of ATP are stored in platelets, the localized release of ATP from activated platelets during PE is highly likely. Although it can be argued that the ubiquitous presence of ecto-enzymes, which rapidly degrade ATP, would preclude the activation of vagal sensory terminals in the left ventricle by ATP originated in the lungs, our previous studies have clearly shown that if enough ATP reaches the right side of the heart, a potent cardio-cardiac vagal reflex is triggered by ATP in the left side of the heart [44]. This is supported by the fact that intravenous, intra-sinus nodal artery or intra-right atrium ATP does not activate vagal sensory terminals in the right side of the heart (e.g., see [45, 46]).

An important related issue is the association between COPD exacerbation [47] and PE, which is relatively high (19.9 and 16.1%) [48, 49]. Platelets express P2Y1R, P2Y12R and P2X1R; the first two are activated by ADP, a metabolite of extracellular ATP and P2X1R is activated by ATP. The activation of these three receptors by extracellular ADP and ATP trigger and amplify platelets’ coagulation [50]. Thus, since abnormally high levels of extracellular ATP are found in the lungs of patients with COPD [24], relatively high frequency of PE in COPD exacerbation is not surprising.

## Conclusions

The data summarized above strongly suggest that ATP released from activated platelets during PE plays a mechanistic role in syncope and bradycardia associated with PE. The P2X3R and/or P2X2/3R and vagal sensory terminals in the lungs and heart could mediate the relevant effects of ATP. Further studies are required to test this hypothesis, which constitutes a potential explanation for these phenomena.

Potential therapeutic guidelines: An established mechanistic role of P2X3R and/or P2X2/3R in this pathophysiological setting would constitute a strong rationale for the use of P2X3R-P2X2/3R antagonists such as Gefapixant, which are currently being developed as drug-candidates for the treatment of COPD and chromic cough [24], may also be indicated after suitable clinical trials in patients with PE, either as an acute treatment for bradycardia and syncope but also as a preventive treatment once the diagnosis of PE has been established.

## Abbreviations

ADP: Adenosine 5′-diphospahte
ATP: Adenosine 5′-triphosphate
COPD: Chronic obstructive pulmonary disease
IgE: Immunoglobulin E
PE: Pulmonary embolism
R: Receptor
